# Screening and regulatory mechanism exploration of M1 macrophage polarization and efferocytosis-related biomarkers in coronary heart disease

**DOI:** 10.3389/fcvm.2024.1478827

**Published:** 2024-12-05

**Authors:** Hong Gao, Junhua Li, Jianxin Huang, Xiaojie Jiang

**Affiliations:** Department of Cardiology, The First Hospital of Nanchang, Nanchang, China

**Keywords:** coronary heart disease, macrophage polarization, efferocytosis, C5orf58, CTAG1A

## Abstract

**Background:**

Macrophage polarization and efferocytosis have been implicated in CHD. However, the underlying mechanisms remain elusive. This study aimed to identify CHD-associated biomarkers using transcriptomic data.

**Methods:**

This study examined 74 efferocytosis-related genes (ERGs) and 17 M1 macrophage polarization-related genes (MRGs) across two CHD-relevant datasets, GSE113079 and GSE42148. Differential expression analysis was performed separately on each dataset to identify differentially expressed genes (DEGs1 and DEGs2). The intersection of upregulated and downregulated genes from both sets was then used to define the final DEGs. Subsequently, MRG and ERG scores were calculated within the GSE113079 dataset, followed by weighted gene co-expression network analysis (WGCNA) to identify key module genes. The overlap between these module genes and the DEGs yielded candidate biomarkers, which were further evaluated through machine learning, receiver operating characteristic (ROC) curve analysis, and expression profiling. These biomarkers were subsequently leveraged to explore immune infiltration patterns and to construct a molecular regulatory network. To further validate their expression, quantitative reverse transcriptase PCR (qRT-PCR) was performed on clinical CHD samples, confirming the relevance and expression patterns of these biomarkers in the disease.

**Results:**

A total of 93 DEGs were identified by intersecting the upregulated and downregulated genes from DEGs1 and DEGs2. WGCNA of the MRG and ERG scores identified 15,936 key module genes in the GSE113079 dataset. Machine learning and ROC analysis highlighted four biomarkers: C5orf58, CTAG1A, ZNF180, and IL13RA1. Among these, C5orf58, and ZNF180 were downregulated in CHD cases, while CTAG1A and IL13RA1 was upregulated. qRT-PCR results validated these findings for C5orf58, CTAG1A, ZNF180, and IL13RA1 showed inconsistent expression trends. Immune infiltration analysis indicated IL13RA1 all had a positive correlation with M0 macrophage, while had a negative correlation with. NK cells activated. The molecular regulatory network displayed that GATA2 and YY1 could regulate CTAG1A and ZNF180.

**Conclusions:**

These results suggest that C5orf58, CTAG1A, ZNF180, and IL13RA1 serve as biomarkers linking M1 macrophage polarization and efferocytosis to CHD, providing valuable insights for CHD diagnosis and therapeutic strategies.

## 1 Introduction

Coronary Heart Disease (CHD), a significant contributor to global cardiovascular mortality, specifically refers to myocardial ischemia, hypoxia, or necrosis due to coronary atherosclerosis (1–3). World Health Organization data indicate that cardiovascular disease claims approximately 17 million lives annually, with CHD accounting for over half of these deaths (4, 5). By 2030, this number is projected to reach 23 million. The pathogenesis of CHD is primarily driven by the buildup of cholesterol and other lipids in the arterial intima, forming atherosclerotic plaques that cause vascular lumen narrowing or blockage. Modifiable risk factors include hypertension, dyslipidemia, diabetes, obesity, smoking, and being overweight, while non-modifiable ones include age and genetic predisposition (6). Current treatments encompass pharmacotherapy, surgical interventions, and lifestyle changes. Pharmacotherapy, often a first-line approach, typically involves antiplatelet, antithrombotic, and antihypertensive agents (7, 8). However, these treatments may have adverse effects and limited effectiveness. While coronary interventions and coronary artery bypass grafting offer substantial benefits, challenges remain, including surgical risks, postoperative recovery, and high costs (9, 10). Consequently, the need for novel diagnostic and therapeutic biomarkers is pressing, as they could enhance the precision of CHD assessment, enable personalized treatment plans, and ultimately improve patient outcomes.

Macrophage polarization refers to the differentiation of macrophages into distinct subpopulations with varying functions and phenotypes in response to specific stimuli (11–15). M1 macrophages primarily drive inflammatory responses, while M2 macrophages are involved in tissue repair and anti-inflammatory processes (16, 17). Efferocytosis, a critical process in which macrophages identify and remove apoptotic cells, maintains intracellular environmental stability (18). This mechanism is essential for preventing the release of toxic cellular contents, promoting tissue regeneration, and averting autoimmune reactions (19). Both macrophage polarization and efferocytosis are governed by intricate regulatory networks involving multiple signaling pathways and cytokine interactions. These processes are central not only to immune regulation and inflammation but also to the pathogenesis of cardiovascular diseases such as atherosclerosis (20–23). Research indicates that M1 macrophages are activated in the early stages of atherosclerosis, where they exacerbate inflammatory responses by releasing pro-inflammatory mediators and chemokines (24, 25). Simultaneously, impaired efferocytosis contributes to atherosclerosis by failing to adequately clear apoptotic cells, leading to necrotic core formation (26). In addition, some scholars have reported that epigenetic regulation contributes to the pathophysiology of cardiovascular disease (CVD) by altering gene expression and controlling various cellular activities, including macrophage polarization (27). Increasing attention has been focused on the interplay between macrophage polarization and efferocytosis in CHD research (11). According to reports, macrophages also produce vascular endothelial growth factor C (VEGFC) through phagocytosis, which can alleviate heart damage and inhibit inflammation, promote lymphatic vessel formation and phagocytosis of cell debris, while inhibiting excessive macrophage secretion of inflammatory factors, thereby playing a role in heart repair (28). Numerous studies have established that both processes are intimately involved in CHD's onset and progression, and their regulation may offer novel therapeutic strategies (29–32). However, despite extensive research, the precise mechanisms underlying macrophage polarization and efferocytosis in CHD remain incomplete.

This study employs comprehensive bioinformatics techniques, utilizing CHD-related transcriptomic data to investigate the potential roles of M1 macrophage polarization and efferocytosis in CHD development. Differential expression analysis was applied to assess changes in gene expression related to these processes. Machine learning algorithms were then employed to identify biomarkers tightly linked to CHD pathology. Enrichment analysis and other methodologies were used to elucidate the regulatory functions of these biomarkers within biological processes and signaling pathways. These analyses aim to deepen the understanding of M1 macrophage polarization and efferocytosis in CHD while identifying potential molecular targets and biomarkers for future therapeutic strategies.

## 2 Methods

### 2.1 Source of data

The Gene Expression Omnibus (GEO, http://www.ncbi.nlm.nih.gov/geo/) database provided two CHD-relevant transcriptome datasets: GSE113079 (GPL20115) and GSE42148 (GPL13607). Basic information for these two datasets was provided in Supplementary Table S1 and Table S2. GSE113079 comprised peripheral blood mononuclear cells (PBMCs) from 93 patients with CHD and 48 controls, while GSE42148 included whole blood samples from 13 CHD cases and 11 controls. A comprehensive literature review was also performed to identify genes linked to M1-type macrophage polarization and apoptotic cell clearance. This review prioritized recent peer-reviewed studies published in high-impact journals, focusing on research involving macrophage polarization and efferocytosis with available gene expression data. Excluded were conference abstracts, case reports, non-human studies, and incomplete data. From the MsigDB database (https://www.gsea-msigdb.org/gsea/msigdb), 35 macrophage polarization-related genes were identified, which were then annotated and categorized into M1 and M2 subtypes. Seventeen M1 macrophage polarization-related genes (MRGs) were selected for further analysis. Additionally, redundant genes were removed from the list of efferocytosis-related genes (ERGs), resulting in a final set of 74 ERGs (33, 34) (Supplementary Table S3).

### 2.2 Differential expression analysis

In order to distinguish differentially expressed genes obtained from the two datasets, we defined the differentially expressed genes (DEGs) derived from dataset GSE113079 as DEGs1, and from the dataset GSE42148 as DEGs2. The GSE113079 and GSE42148 datasets were analyzed using the limma package (v 3.52.4) (35) to identify differentially expressed genes (DEGs1 and DEGs2) with |log2FC|>0.5 and *P* < 0.05. Volcano and heatmaps were generated using ggplot2 (v 3.3.6) (36) and pheatmap (v 1.0.12) (37) to visualize DEGs1 and DEGs2. The intersecting upregulated and downregulated genes from both datasets were merged to generate contro vs. case DEGs.

### 2.3 Weighted gene co-expression network analysis (WGCNA)

To identify CHD-associated genes linked to MRGs and ERGs, single-sample gene set enrichment analysis (ssGSEA) in the GSVA package (v 1.44.5) (38) was used to calculate the ssGSEA enrichment scores for the samples included in the GSE113079, usingMRGs and ERGs as background sets. WGCNA, as a powerful bioinformatics tool, is specifically used to excavate gene modules closely linked to particular diseases, thereby further unveiling the core genes within these modules. In this process, “modules” specifically refer to clusters of genes exhibiting highly similar expression characteristics. To decipher the association between gene sets and sample phenotypes, this study constructs a regulatory network among gene sets and identifies key regulatory genes. Firstly,the Wilcoxon test (*P* < 0.05) compared scores between the case and control groups. Next, module genes were identified using weighted gene co-expression network analysis (WGCNA, v 1.72-1) (39), with MRG and ERG scores serving as trait variables. Samples in GSE113079 were initially clustered to exclude outliers, and to ensure gene interactions conformed to a scale-free topology, the *R*^2^ was adjusted to 0.85 with a mean connectivity approaching 0. An optimal soft threshold was selected for module detection, and a dynamic tree-cutting algorithm, requiring a minimum of 100 genes per module, was used to construct the module hierarchy. Pearson correlations between modules and the MRG and ERG scores were independently calculated, with genes from modules showing correlations >0.3 and *P* < 0.05 being selected as key module genes.

### 2.4 Recognition and functional exploration of candidate genes

Candidate genes were selected from the intersection of DEGs and key module genes. These candidates underwent Gene Ontology (GO) and Kyoto Encyclopedia of Genes and Genomes (KEGG) enrichment analysis using the clusterProfiler package (v 4.7.1) (40) to explore their involvement in biological processes (*P* < 0.05). The top 10 enriched terms were visualized using a tree diagram generated by the treemap package (v 2.4-4) (41).

### 2.5 Machine learning

To identify feature genes linked to macrophage polarization and efferocytosis, machine learning techniques were applied to the candidate genes. First, Support Vector Machine Recursive Feature Elimination (SVM-RFE) was conducted using e1071 (v 1.7-12) (42) to identify the optimal combination of genes with the lowest error rate, thereby extracting relevant feature genes. Concurrently, a 3-fold cross-validation approach was used with the glmnet package (v 4.1-7) (43) to perform the least absolute shrinkage and selection operator (LASSO) analysis, identifying feature genes corresponding to the minimal Lambda value. Genes simultaneously identified by both SVM-RFE and LASSO were further analyzed through receiver operating characteristic (ROC) curve plotting and expression profiling in both GSE113079 and GSE42148 datasets. Genes exhibiting consistent expression trends and an area under the ROC curve (AUC) greater than 0.8 in both datasets were recognized as biomarkers (*P* < 0.05).

### 2.6 Immune infiltration

To further assess the immune cell composition in CHD, immune infiltration analysis was performed on the GSE113079 dataset using the CIBERSORT program (v 1.03) (44), which estimates the proportions of 22 immune cell types. The Wilcoxon test (*P* < 0.05) was employed to compare immune cell distributions between case and control groups. Spearman's correlation analysis was conducted to evaluate the relationships between biomarkers and differentially expressed immune cells, particularly M0/M1 macrophages.

### 2.7 Construction of molecular regulatory networks

To explore the regulatory mechanisms of the identified biomarkers, transcription factors (TFs) were predicted using the JASPAR database through the NetworkAnalyst 3.0 platform (https://www.networkanalyst.ca/). miRNA targets for the biomarkers were predicted using the miRWalk (http://mirwalk.umm.uni-heidelberg.de) and miRDB (http://www.mirdb.org/) databases. Finally, networks of biomarker-TF and miRNA-biomarker interactions were constructed to elucidate their regulatory roles.

### 2.8 The quantitative reverse transcriptase PCR (qRT-PCR)

To validate biomarker expression via qRT-PCR, peripheral blood samples were collected from five patients with CHD and five healthy individuals at the First Hospital of Nanchang. The patient's clinical information can be found in Supplementary Table S4. PBMCs were extracted by adding 3 ml of whole blood to a 15 ml centrifuge tube containing 3 ml of PBMC isolate. Following centrifugation at 2,000 g for 20 min, the second layer of cells was carefully transferred to a fresh 15 ml centrifuge tube containing 15 ml of phosphate-buffered saline (PBS). After 10-min centrifugation at 1,000 g, the supernatant was discarded, and the cell pellet was mixed thoroughly with 1 ml of TRIzol (Ambion, USA). PCR extraction was then performed, ensuring the cell precipitate was fully homogenized. After RNA quantification, reverse transcription was immediately initiated using the SweScript First Strand cDNA Synthesis Kit (Servicebio, China) following the manufacturer's protocol. qPCR amplification was conducted using a 40-cycle program on the CFX96 Real-Time Fluorescence Quantitative PCR Instrument. Primer sequences are detailed in Table 1, and the 2^−ΔΔCt^ method was used to analyze biomarker expression.

**Table 1:** Primer sequences.

Primer	Sequence
C5orf58 F	GAACCTCGGTGACGGTTGG
C5orf58 R	GAGGGGGTTGTTTCTTTCTGC
CTAG1A F	CTGCAGCCTCTCTGCCTC
CTAG1A R	ACAGTTGCGGCTCAGTAGAG
ZNF180 F	GGAGGAGAGCATGGAAGAGC
ZNF180 R	AAGTACCCTGTTCCTCCCGT
IL13RA1 F	CTTGGCTCTTGTCTGCTGGA
IL13RA1 R	CTCTTCTCCAAAGCGCCCAT
GAPDH F	CGAAGGTGGAGTCAACGGATTT
GAPDH R	ATGGGTGGAATCATATTGGAAC

### 2.9 Statistical analysis

All bioinformatics analyses were performed in R (v 4.3.1). The Wilcoxon test (*P* < 0.05) was applied to compare data between the CHD and control groups.

## 3 Results

### 3.1 Efferocytosis and M1 macrophage polarization were associated with CHD

**Figure 1:**
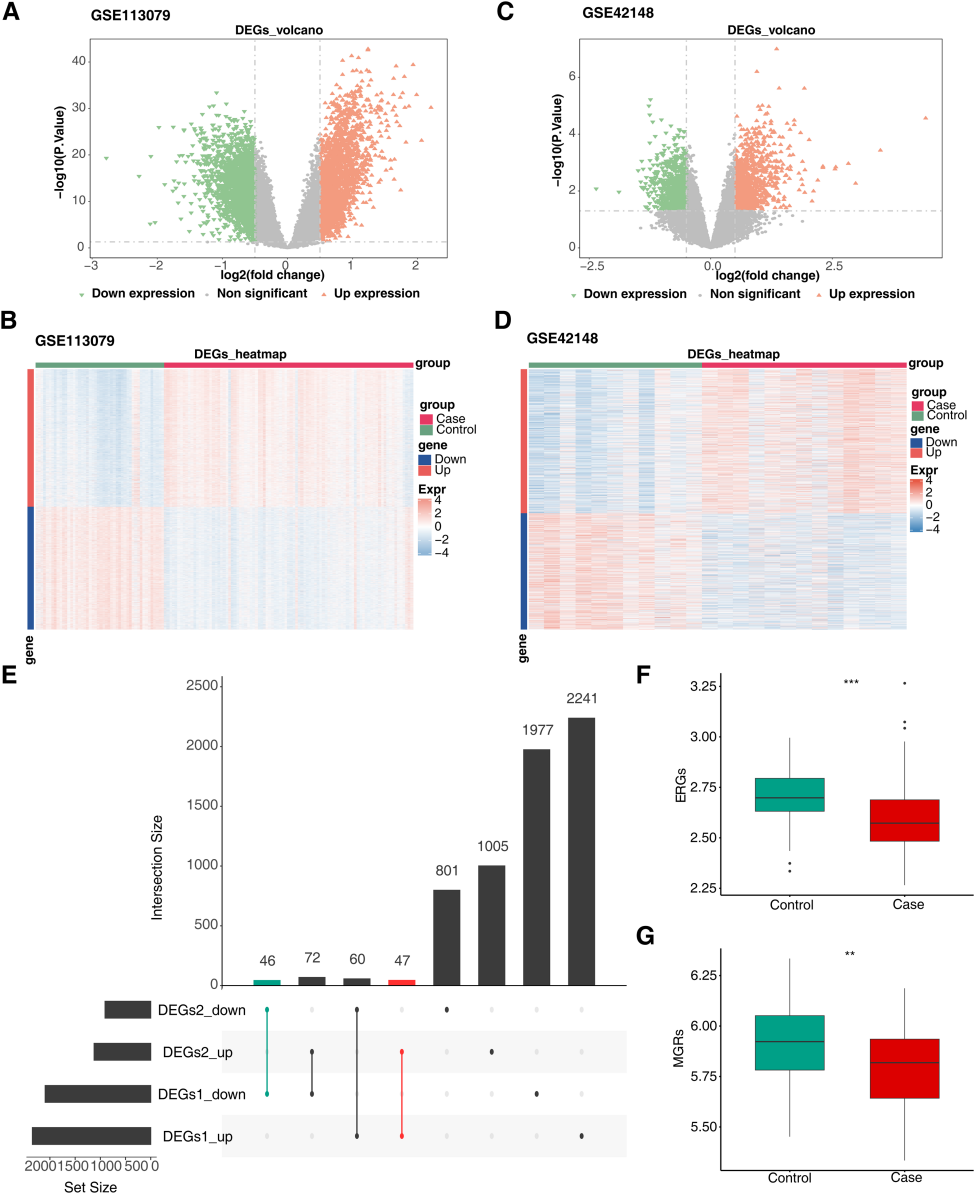
Screen for differentially expressed genes related to efferocytosis and M1 macrophage polarization in CHD. **(A)** Volcano plot of DEGs1. **(B)** Heatmap of DEGs1. **(C)** Volcano plot of DEGs2. **(D)** Heatmap of DEGs2. **(E)** Venn diagram of intersecting DEGs. The left - hand bar chart shows the number of genes in each subset; the upper bar chart shows the number of genes in each intersection; green represents the number of commonly down - regulated genes; red represents the number of commonly up - regulated genes. **(F)** Differences in ERGs scores between groups. **(G)** Differences in MRGs scores between groups. **(A,C)** Each point in the graph represents a gene. Orange represents significantly up - regulated genes, green represents significantly down - regulated genes, and gray represents non - significant genes. **(B,D)** Green represents the Control samples, and red represents the Case samples; in the graph, red indicates highly - expressed genes, and blue indicates low - expressed genes.

Differential expression analysis of the GSE113079 and GSE42148 datasets identified 4,443 DEGs1 (Figures 1A,B; Supplementary Table S5) and 2,031 DEGs2 (Figures 1C,D; Supplementary Table S6), respectively. DEGs1 consisted of 2,348 upregulated and 2,095 downregulated genes, while DEGs2 included 1,124 upregulated and 907 downregulated genes. By intersecting the upregulated and downregulated genes from both datasets, 47 upregulated and 46 downregulated intersecting genes were identified, resulting in a total of 93 DEGs (Figure 1E). Additionally, GSVA scores based on MRGs and ERGs showed significant differences between patients with CHD and controls, with all scores being lower in the disease group, indicating that CHD is associated with both M1 macrophage polarization and efferocytosis (Figures 1F,G).

### 3.2 The genes linked to M1 macrophage polarization and efferocytosis in CHD

**Figure 2:**
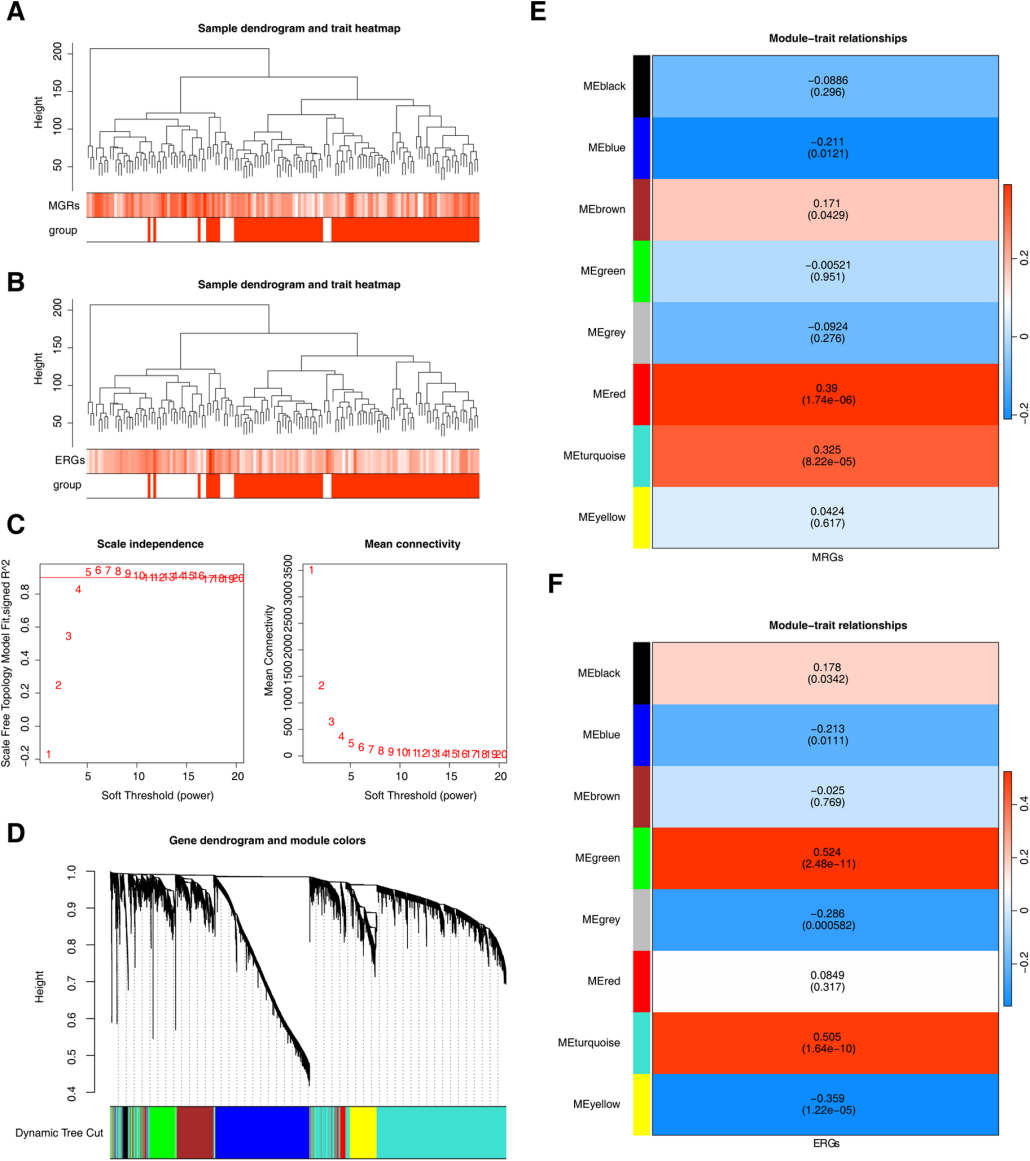
Genes related to M1 macrophage scores and efferocytosis in CHD. **(A)** Clustering of M1 macrophage samples. **(B)** Clustering of efferocytosis samples. **(A,B)** Branches represent samples, and the vertical axis represents the height of hierarchical clustering. The darker the color above, the higher the score, and the red group below represents disease samples. **(C)** Scale-free fit index and mean connectivity analysis for various soft-thresholding powers. The horizontal axis represents the power value of the weight parameter. In the left figure, the vertical axis is the scale - free fit index, that is, signed *R*^2^. The higher the square of the correlation coefficient is, the closer the network is to the scale - free distribution. In the right figure, the vertical axis represents the average value of all gene adjacency functions in the corresponding gene module. **(D)** Dendrogram of co-expression module clustering. Different colors represent distinct co-expression modules. **(E,F)** Heatmap of the correlation between modules and scored traits. It contains a group of highly related genes, and each color indicates a specific gene module.Select modules with an absolute value of correlation greater than 0.3 and *P* less than 0.05. Therefore, select the red, turquoise, and green, turquoise, yellow modules as key modules respectively.

Using MRGs and ERGs genes as background gene sets to obtain enrichment scores, and the findings revealed no outliers (Figures 2A, B). The optimal soft-thresholding power was determined to be 5, using both *R*^2^ and mean connectivity criteria (Figures 2C,D). The hierarchical clustering tree divided all genes in the GSE113079 dataset into eight modules (Figures 2E,F), with the red (*r* = 0.39, *P* = 1.74 × 10^−6^) and turquoise (*r* = 0.33, *P* = 8.22 × 10^−6^) modules showing the highest correlation with M1 macrophage scores (Figure 2E). Similarly, the green (*r* = 0.52, *P* = 2.48 × 10^−11^), turquoise (*r* = 0.51, *P* = 1.64 × 10^−10^), and yellow (*r* = −0.36, *P* = 1.22 × 10^−6^) modules were most strongly correlated with efferocytosis characteristics (Figure 2F). The results showed that we obtained 7,079 genes associated with MRGs and 8,839 gene modules associated with ERGs.

### 3.3 ZNF180, CTAG1A, Il13ra1, and C5orf58 were detected as biomarkers linked to efferocytosis and M1 macrophage polarization

**Figure 3:**
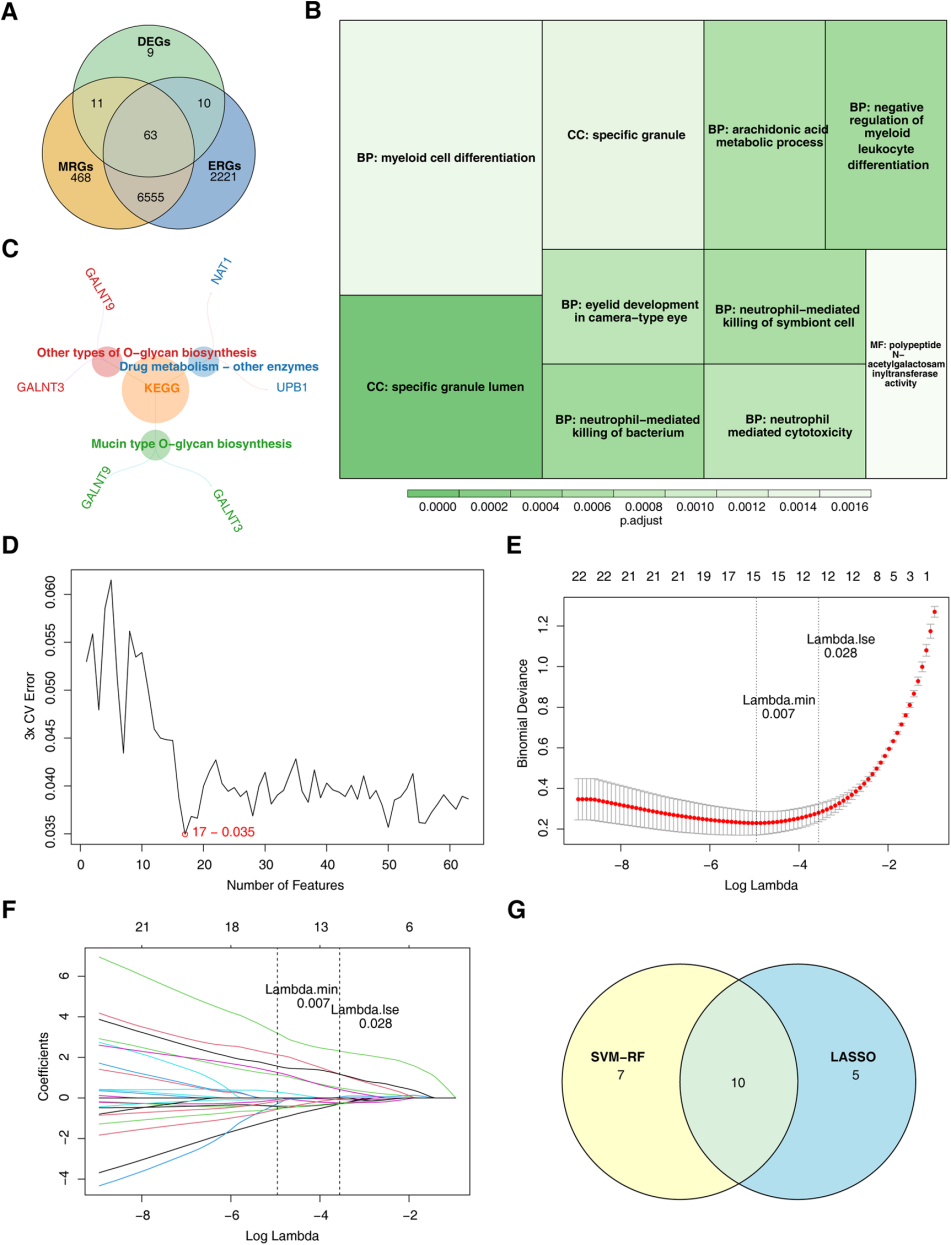
ZNF180, CTAG1A, IL13RA1, and C5orf58 identified as biomarkers related to efferocytosis and M1 macrophage polarization. **(A)** Candidate gene identification. **(B)** GO enrichment results for candidate genes. The size of the square represents the number of enriched genes; the color represents significance. **(C)** KEGG enrichment results for candidate genes. **(D)** SVM-RFE analysis. The abscissa represents the number of genes, and the ordinate represents the error rate **(E,F)** LASSO regression analysis graph. **(E)** The graph of the penalty term parameter. The position of the left - hand dotted line is the position where the cross - validation error is the smallest. Determine log(Lambda) according to this position (lambda.min), and the number of feature genes is shown above. Find the optimal log(Lambda) value, and find the corresponding genes and their coefficients in the right figure; **(F)** After different variables are penalized by *λ*, the changes in their coefficients. **(G)** Genes identified by both machine learning algorithms.

**Figure 4:**
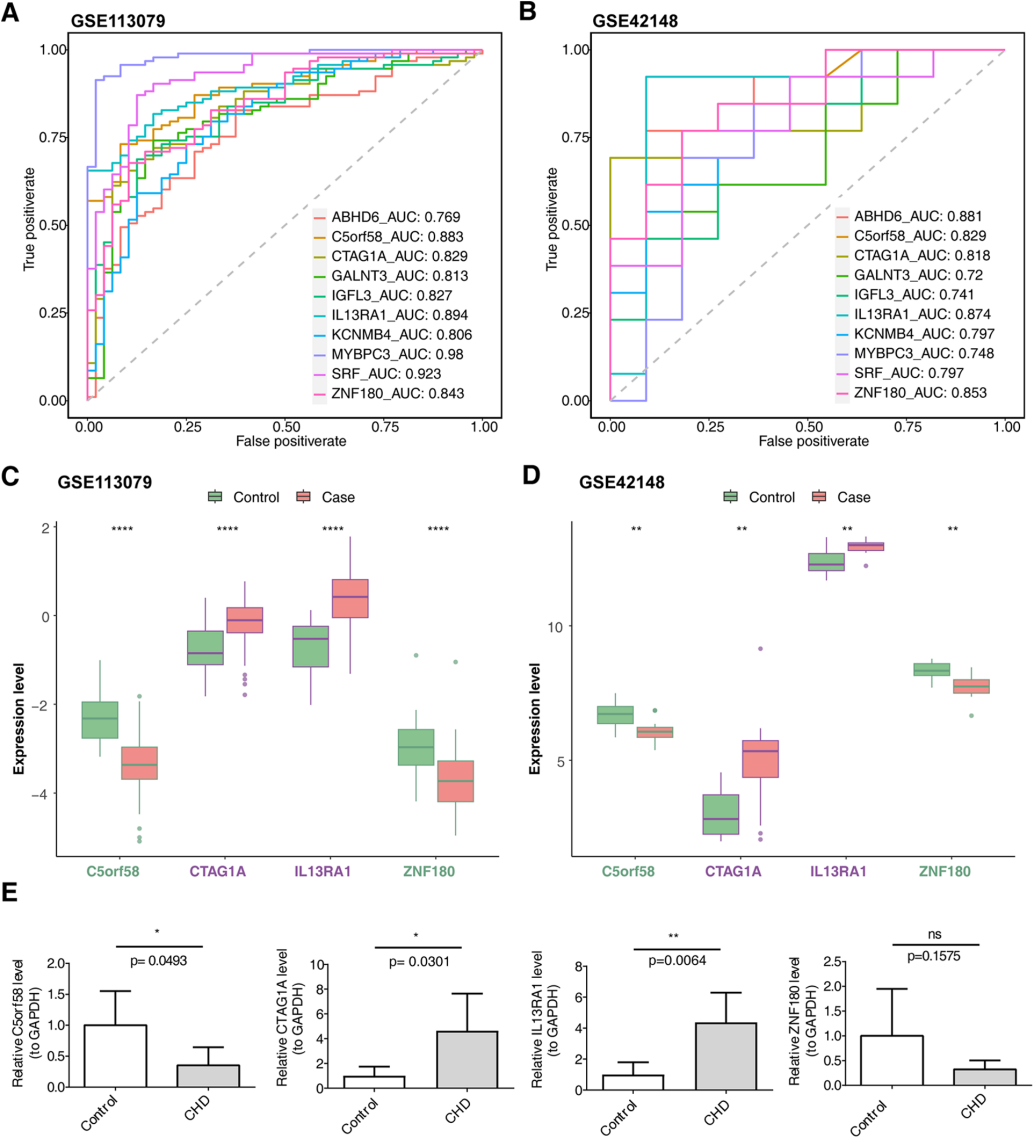
Biomarker expression levels and diagnostic performance evaluation. **(A)** ROC curves for biomarkers in the GSE113079 dataset. **(B)** ROC curves for biomarkers in the GSE42148 dataset. **(A,B)** The abscissa is the false positive rate. The smaller X is, the higher the accuracy rate. The ordinate is the true positive rate. The larger Y is, the higher the accuracy rate. By evaluating the true positives and false positives of different thresholds, a curve can be constructed. This curve extends from the lower left to the upper right and bends towards the upper left. A classifier with no discriminative power between positive and negative classes will form a diagonal line, with the two ends being (0, 0) and (1, 1) respectively. **(C)** Intergenomic expression status in the GSE113079 dataset. **(D)** Intergenomic expression status in the GSE42148 dataset. **(C,D)** The abscissa represents genes, and the ordinate represents the expression level; the box color indicates sample grouping; the abscissa color indicates gene grouping (purple represents up - regulation, green represents down - regulation, and black represents non - significant), and the top shows significance, *, *p* < 0.05; **, *p* < 0.01; ***, *p* < 0.001. **(E)** Differential expression of biomarkers in patients with CHD and controls based on qRT-PCR. The sample size was 5.

The intersection of key module genes and DEGs yielded 63 candidate genes (Figure 3A). Functional enrichment analysis (Supplementary Table S7) revealed that these genes were primarily associated with 244 GO functions, such as neutrophil-mediated bactericidal activity, specific granule lumen, and polypeptide N-acetylgalactosaminyltransferase activity, along with three KEGG pathways: mucin-type O-glycan biosynthesis, other types of O-glycan biosynthesis, and drug metabolism – other enzymes (Figures 3B, C). From these candidate genes, the SVM-RFE model identified 17 feature genes with the lowest error rate (Figure 3D). Meanwhile, LASSO regression analysis revealed that the minimal error rate occurred when Lambda was set at 0.007, identifying 15 feature genes (Figures 3E, F). After screening, a total of 10 feature genes were identified by both machine learning methods: ABHD6, C5orf58, CTAG1A, GALNT3, IGFL3, IL13RA1, KCNMB4, MYBPC3, SRF, and ZNF180 (Figure 3G). ROC curve analysis demonstrated that C5orf58, CTAG1A, ZNF180, and IL13RA1 had AUC values exceeding 0.8 in both the GSE113079 and GSE42148 datasets, indicating that these four genes were strong predictors of CHD (Figures 4A, B). Meanwhile, C5orf58 and ZNF180 were all downregulation in case group, whilst CTAG1A and IL13RA1 upregulation in expression, establishing C5orf58, CTAG1A, ZNF180, and IL13RA1 as biomarkers (Figures 4C, D). The qRT-PCR results indicated that the all biomarkers had expression trends similar to those in the datasets (Figure 4E).

### 3.4 The naive Cd4t cells and M0 macrophages might regulate CHD by interacting with biomarkers

**Figure 5:**
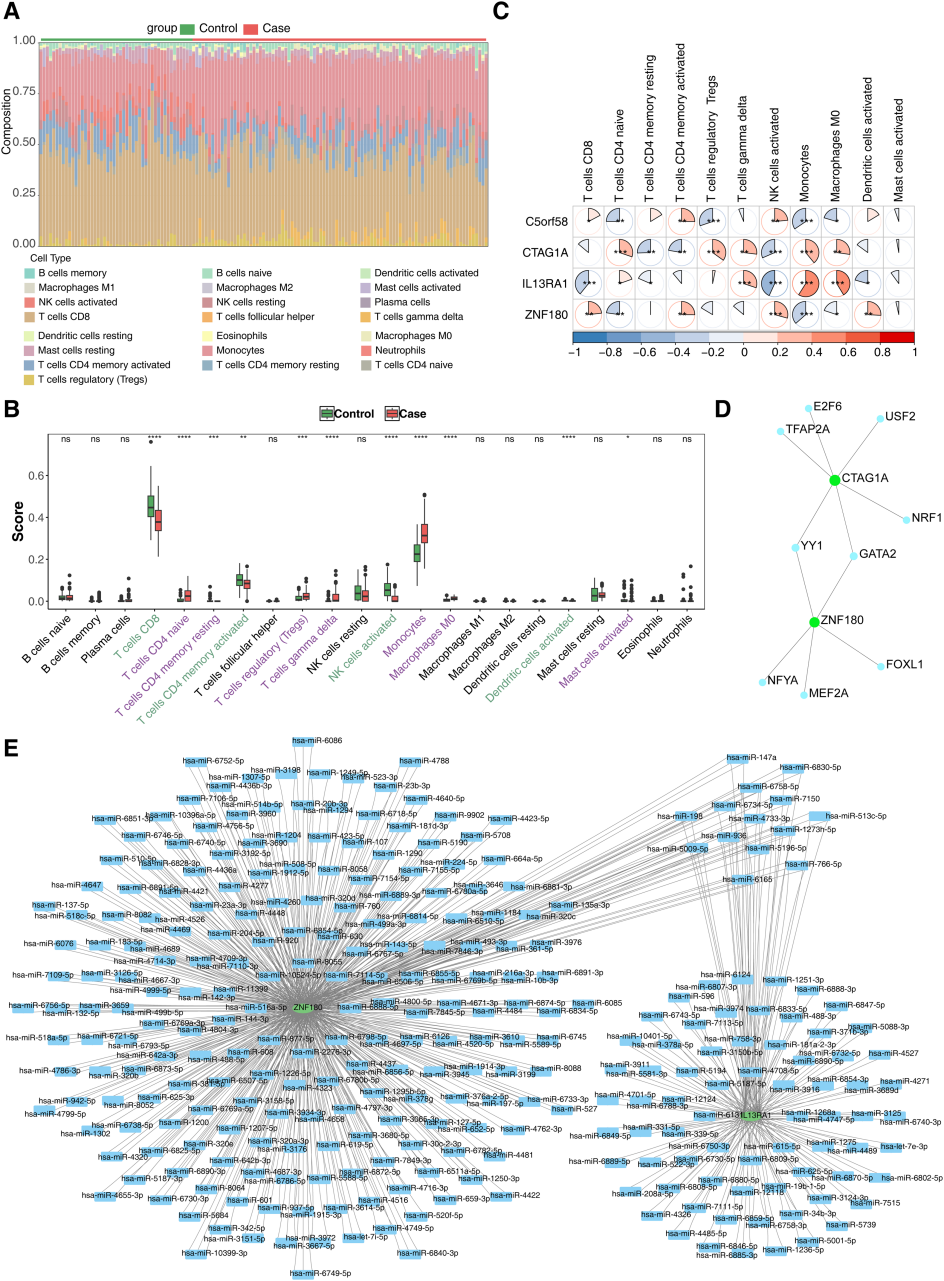
Immune infiltration analysis and molecular regulation analysis. **(A)** Stacked diagram of immune cell infiltration. **(B)** Box plot of immune cell infiltration situation. The abscissa represents 22 kinds of immune cells (purple indicates significantly up - regulated immune cells, and green indicates significantly down - regulated immune cells), and the ordinate represents the scores of immune cells in the samples. ns, not significant; *, *P* < 0.05; **, *P* < 0.01; ***, *P* < 0.001. **(C)** Heat map of biomarkers and differential immune cell correlation. Red indicates a positive correlation, blue indicates a negative correlation; significance: *, *P* < 0.05; **, *P* < 0.01; ***, *P* < 0.001 **(D)** TF-biomarkers regulatory network;Key genes are shown in green and transcription factors in blue. **(E)** Construction of miRNA-biomarkers network. The mRNA is shown in green, and the predicted miRNA is shown in blue.

To investigate the role of immune cells in CHD, an immune cell infiltration analysis was performed. The proportion of immune cells in the case and control samples is displayed in Figure 5A. Differential expression analysis of these immune cells revealed significant differences in 10 immune cell types between the two groups. Specifically, CD8T cells, memory-activated CD4T cells, activated NK cells, and activated dendritic cells were significantly downregulated in the case group, while the remaining immune cells exhibited opposite trends (Figure 5B). Finally, the correlation analysis of biomarkers with immune cells showed that Monocytes had the strongest positive (*r* = 0.330, *p* < 0.001) with IL13RA1, NK cells had strongest negative (*r* = -0.432, *p* < 0.001) with IL13RA1 (Figure 5C).

### 3.5 The regulation of biomarkers was mediated by a number of molecules, GATA2 potentially being one of them

To further explore the molecular regulatory mechanisms of these biomarkers in CHD, TF prediction was carried out. The results showed that only CTAG1A and ZNF180 had transcription factors predicted, while the other two biomarkers did not have transcription factors predicted. A total of 9 transcription factors were predicted. Among them, YY1 and GATA2 have regulatory effects in both CTAG1A and ZNF180, indicating that they may have a regulatory functions in CHD. (Figure 5D). Meanwhile, 710 and 206 miRNAs targeting biomarkers were estimated in the miRWalk and miRDB databases, accordingly. Only two key genes were predicted in the database. This network has 294 nodes and 803 pairs of interaction relationships (Figure 5E).

## 4 Discussion

CHD, as a prevalent global cardiovascular disorder, poses a significant threat to human health. Its pathogenesis is multifaceted, involving the interplay of various cellular and molecular mechanisms. In recent years, growing evidence has underscored the critical role of M1 macrophage polarization and its association with efferocytosis in the onset and progression of CHD (28, 45–48). This study employed differential gene expression analysis to identify changes in the expression of genes related to M1 macrophage polarization and efferocytosis in CHD, providing preliminary insights into their potential involvement in the disease. Through machine learning techniques, this study further identified biomarkers (C5orf58, CTAG1A, ZNF180, and IL13RA1) that are closely associated with CHD pathology, suggesting their potential as therapeutic targets. Enrichment analysis and other approaches were then applied to elucidate the regulatory roles of these biomarkers in key biological functions and signaling pathways, offering valuable insights into the role of M1 macrophage polarization and efferocytosis in CHD.

Cancer/Testis Antigen 1A (CTAG1A) is a protein coding gene associated with cancer and is a member of the Cancer Testis Antigen (CTA) family. At present, there are relatively few studies on CTAG1A, which highlights the innovation of our research. Research has shown that CTAs have high tumor specificity and sensitivity (49, 50), making them considered tumor specific biomarkers and potential targets for cancer treatment. In this study, CTAG1A, as one of the cancer/testicular antigen related genes, was screened as a biomarker for coronary heart disease. We speculate that it may play an important regulatory role in the occurrence and development of coronary heart disease.

IL13RA1 (interleukin-13 receptor subunit alpha 1) belongs to protein coding genes. The protein encoded by this gene is a subunit of the interleukin-13 receptor. Currently, research on IL13RA1 is relatively scarce, which highlights the innovation of our study. Interleukin-13 (IL-13) is a cytokine with multiple functions in the immune system, acting through the IL-13 receptor (IL-13R) (51). There are research reports that the immune system plays a key role in the process of cardiac homeostasis and failure in humans and mice through IL-13R α 1 signaling (52). This indicates that the biomarker IL13RA1 identified in this study may play an important regulatory role in the occurrence and development of coronary heart disease.

ZNF180 is a protein coding gene belonging to the ZNF family. According to reports, the expression of this gene is downregulated due to methylation in colorectal cancer. *In vitro* and *in vivo* experiments have shown that overexpression of ZNF180 is functionally associated with inhibiting cell proliferation and inducing cell apoptosis (53). In addition, ZNF180 is a coding gene that regulates immune cell infiltration in melanoma cells and is negatively correlated with the expression of plasminogen activator inhibitor-1 (54). There are also studies indicating that ZNF180 is a candidate protein for coronary artery disease (CAD) (55), suggesting that it may be a potential drug target for CAD. At present, research on ZNF180 is relatively limited, and there have been no reports on coronary heart disease, which highlights the innovation of this study and the novelty of the results obtained. C5orf58 (open reading frame 58 on chromosome 5) was found to be highly methylated and expressed at low levels in hepatocellular carcinoma. However, the biological effects of C5orf58 had not been reported prior to this. According to Entrez Gene, C5orf58 is located on chromosome 5q35.1. This study is the first to investigate C5orf58, demonstrating the innovation of this research. This study reveals for the first time the correlation between C5orf58, ZNF180, M1 macrophage polarization, and increased bubble cells in coronary heart disease. The qRT PCR results of key genes are consistent with the expression trends in the dataset. Although the *P*-value of ZNF180 was greater than 0.05 in the experiment, due to the fact that the entire study was based on significantly differentially expressed genes, ZNF180 was significantly expressed in our processed samples and was also significant in dataset validation. According to reports, the expression of ZNF180 also showed significant differences in coronary artery disease (CAD) (55), and the disease studied in this study belongs to the CAD type, indicating that ZNF180 plays an important role in coronary heart disease. The insignificant qRT PCR results in this study may be due to factors such as small sample size, data analysis, or experimental procedures. In future research, we will increase the sample size and optimize experiments to improve the reliability of statistics.

In recent years, the immune-inflammatory mechanism has been recognized as a key factor in the pathogenesis of CHD. Macrophages, key players in innate immune memory, are critically involved in atherogenesis by promoting the release of inflammatory cytokines, enhancing cholesterol deposition, and disrupting vascular smooth muscle cell function. Macrophage-targeted therapies have thus emerged as a potential strategy for CHD treatment. Similarly, T cell subsets, including CD4^+^ T cells and CD8^+^ T cells, contribute significantly to CHD by stimulating immune responses, producing inflammatory cytokines, promoting lipoprotein accumulation, and inducing plaque formation (56). Our immune infiltration and Spearman correlation analysis showed significant differences in 10 immune cells, including macrophages and T cells, between the coronary heart disease group and the control group. Meanwhile, CTAG1A and IL13RA1 were positively correlated with macrophage M0, and the correlation was significant, while ZNF180 and C5orf58 were negatively correlated. And the correlation is not significant. In addition, the correlation between monocytes and these four genes is very significant. Among them, monocytes are positively correlated with CTAG1A and IL13RA1, and negatively correlated with ZNF180 and C5orf58.These results not only deepen our understanding of CHD's immune mechanisms but also offer valuable insights for developing targeted treatment strategies.

GO and KEGG enrichment analyses were conducted to explore candidate gene interactions in greater depth. The results identified that these genes were primarily involved in 244 GO functions, such as neutrophil-mediated bacterial killing, specific granule cavities, and peptide N-acetylglucosamine transferase activity. Research has found that there are 19 members of the peptide-N-acetylglucosamine transferase activity family in mice. Galnt1 is essential for normal heart development and function, and its gene deletion can lead to abnormal valve development and heart function (57); The deletion of galnt11 gene can lead to congenital heart disease and visceral ectopia (58). This indicates that our enrichment results are related to coronary heart disease. Additionally, they were associated with three key KEGG pathways: mucin-type O-polysaccharide biosynthesis, other types of O-polysaccharide biosynthesis, and drug metabolism by other enzymes. These findings strongly suggest that the candidate genes play a significant role in cellular immune regulation. Notably, previous studies have shown that Th17 cell infiltration occurs from the early to late stages of coronary atherosclerosis. Neutralizing IL-17A blocked atherosclerosis progression in Apoe -/- mice on a high-fat diet, and inhibiting CD4^+^ T cell polarization slowed coronary disease progression (59). Furthermore, homocysteine-activated CD4^+^ T cells were found to increase pyruvate kinase muscle isoenzyme 2 (a key rate-limiting enzyme in glycolysis), thereby accelerating arteriocoronary atherosclerosis (60). The ROC curve analysis of this study confirms that the four biomarkers C5orf58, CTAG1A, ZNF180, and IL13RA1 all exhibit good diagnostic performance, with AUC values exceeding 0.8. This discovery is consistent with the previous research results of Guo Liwei et al., further strengthening these genes as reliable biomarkers for coronary heart disease. These results not only offer new avenues for early CHD diagnosis but also provide a foundation for future therapeutic strategies.

This study focuses on the roles of C5orf58, ZNF180, CTAG1A, and IL13RA1 in the polarization of M1 macrophages and the increase of bubble cells in coronary heart disease. Through systematic immune correlation analysis and molecular regulation predictions, these biomarkers were revealed to have a potentially significant impact on CHD pathogenesis, particularly in relation to M1 macrophage polarization. This research contributes new insights into the pathophysiological mechanisms of CHD and establishes a theoretical framework for developing novel therapeutic strategies. Although our research has achieved good results, the limitation of sample size is a factor that cannot be ignored. The limited sample size increases the uncertainty of the results. Therefore, our results are only preliminary and require more extensive data to validate. Given the limitations of sample size, future research should consider increasing the sample size to improve statistical power and the reliability of results. Consider collaborating through multiple centers to gather data from different institutions to achieve sufficient sample size. Meanwhile, functional experiments targeting key genes, such as knockout or overexpression experiments, have not been implemented in current research due to limitations in experimental conditions and techniques. However, future research should focus on overcoming these technological barriers and adopting more advanced methods, such as CRISPR/Cas9 gene editing technology, to delve deeper into gene function. This is the only way to more accurately evaluate the role of genes in disease development and their potential as therapeutic targets.

## 5 Conclusions

This study focuses on four biomarkers C5orf58, ZNF180, CTAG1A, and IL13RA1 that are associated with M1 macrophage polarization and bubble cell proliferation in coronary heart disease, highlighting their key roles in the pathogenesis of the disease. Immune correlation and molecular regulation analyses offer fresh insights into the underlying pathophysiology of coronary heart disease, establishing a theoretical basis for developing therapeutic strategies. Additionally, the findings underscore the significance of immune regulation in the progression of the disease and emphasize the diagnostic potential of these biomarkers.

## Data Availability

Publicly available datasets were analyzed in this study. This data can be found below. The datasets analyzed during the current study are available in the Gene Expression Omnibus (GEO) repository [http://www.ncbi.nlm.nih.gov/geo/], accession numbers GSE113079 and GSE42148; JASPAR database [https://www.networkanalyst.ca/], transcription factors include C50rf58, CTAG1A, IL13RA1 and ZNF180 (please note that this database is an online resource and does not have a unique access code); Overview of the miRWalk database [http://mirwalk.umm.uni-heidelberg.de]: an online resource, ‘miRWalk’ covers genes such as C50rf58, CTAG1A, IL13RA1 and ZNF180. Again, since it is an online database, there is no login code available; information on the miRDB database [http://www.mirdb.org/]: the miRDB is also an online platform for the same list of genes: C50rf58, CTAG1A, IL13RA1, ZNF180, etc. As mentioned above, no login code is required to access this database.
